# Complete genome sequence of soybean vein necrosis virus (SVNV) from Alabama

**DOI:** 10.1128/mra.00498-25

**Published:** 2025-09-05

**Authors:** Abdelaal H. A. Shehata, Rachel Livingston, Neha Potnis, Edward J. Sikora, K. M. Martin

**Affiliations:** 1 Department of Entomology and Plant Pathology, Auburn University 1383, Auburn, Alabama, USA; 2 Department of Plant Pathology, Faculty of Agriculture, Assiut University 1383, Assiut, Egypt; Katholieke Universiteit Leuven, Leuven, Belgium

**Keywords:** soybean vein necrosis virus, full genome, Alabama isolate, RNA sequencing, SNPs, indels, RACE

## Abstract

The complete genome of soybean vein necrosis virus (SVNV) isolate SVNV17_Auburn_AL was obtained from soybean using RNA sequencing and rapid amplification of cDNA ends (RACE). The tripartite genome comprises 16,563 nucleotides, representing the Alabama (AL) SVNV genome, and shows high similarity to Iowa, Illinois, and Tennessee isolates, enabling evolutionary analysis.

## ANNOUNCEMENT

Soybean vein necrosis virus (*Orthotospovirus glycininecrovenae*, SVNV) is an ambisense ssRNA virus in the genus *Orthotospovirus* first identified in Tennessee in 2008 (1). SVNV consists of three segments: S, M, and L. These encode a nucleocapsid protein (N), nonstructural proteins (NSs and NSm), glycoproteins (G_N_ and G_C_), and an RNA-dependent RNA polymerase (RdRp) (2). The complete sequence of the SVNV17_Auburn_AL isolate was obtained using RNA-Seq and RACE.

In 2023, soybean samples exhibiting symptoms of SVNV were collected. Total RNA was extracted from symptomatic leaves using the previous methodology (3), followed by ribosomal RNA depletion using the Illumina Ribo-Zero Plus rRNA Depletion Kit (Illumina, Cat: 20037135). Libraries were prepared with the NEBNext Ultra II Directional RNA Library Prep Kit for Illumina and sequenced on an Illumina NovaSeq 6000 (150 bp PE, ~47 million reads). Quality control was conducted using FastQC (4), and adapter sequences were removed using BBDuk (https://sourceforge.net/projects/bbmap/). Processed reads were mapped to the SVNV-TN genome (GCA_004789395.1) using Bowtie (5). Variants (depth >80, Phred > 100) were called using BCFtools (6) and FreeBayes tool (7), and the average depth was calculated using SAMtools (6). Identified variants were visualized using IGV v2.3.57 (8). The consensus assembly was generated with BCFtools (6). For all tools, the default parameters were used. Missing terminal nucleotides were filled using RACE: 5′ ends with the Invitrogen 5′ RACE System (ThermoFisher, Cat: 18374058) using segment-specific primers (Table 1), and 3′ ends using *E. coli* poly(A) polymerase (NEB) followed by SuperScript III (Invitrogen) synthesis. PCR amplification used Phusion (ThermoFisher, F530S), cloned using the CloneJET PCR Cloning Kit, and 12 colonies were Sanger sequenced using an Applied Biosystems 3730xl sequencer.

**TABLE 1:** Soybean vein necrosis virus segment-specific primers and PolyT-R, used for rapid amplification of 5′ and 3′ RACE protocols

No	Primer ID	Sequence	Amplified contig	Usage
1	SVNV-S-NSs-300-R	CCTGGGAAAACTTCATCGCTCGACAT	300 bp	5′ - cDNA^*a*^
2	SVNV-S-NSs-274-R	GTCGACAATAGTATCTGGATCCTCAA	274 bp	5′ - PCR^*a*^
3	SVNV-S-N-2329-F	CAATAGCCTTTCTGTTTTTGAGAAAG	274 bp	3′ - PCR
4	SVNV-M-NSm-300-R	TGTACCAGACTCATATACACCTAATT	300 bp	5′ - cDNA^*a*^
5	SVNV-M-NSm-274-R	CAGATGATCCTGTAGTGTCTCTCACA	274 bp	5′ - PCR^*a*^
6	SVNV-M-Gn-4682-R	ACCAAGCTTTTGCCAATTTTTCCTTG	274 bp	3′ - PCR
7	SVNV-L- RdRp-300-R	GAGTAGTTCAGTTGGAAACTAGTTCA	300 bp	5′ - cDNA^*a*^
8	SVNV-L- RdRp-274-R	AGTGGTTCGAAAGCTAAATGGTCAAA	274 bp	5′ - PCR^*a*^
9	SVNV-L-RdRp-8737-F	CTTCAACTTTAACATGGTTGTAGATT	274 bp	3′ - PCR
10	PolyT-R	TTTTTTTTTTTTTTTTTTTTTTTTTTTTTT	NA	3′ - PCR

^*a*^ Primers marked with an asterisk were used for the 5′ end following the RACE protocol instructions: the first primer (labeled “cDNA”) was used for first-strand cDNA synthesis, and the second primer located slightly downstream of the first primer (labeled “PCR”) was used for subsequent PCR amplification.

**Fig 1:**
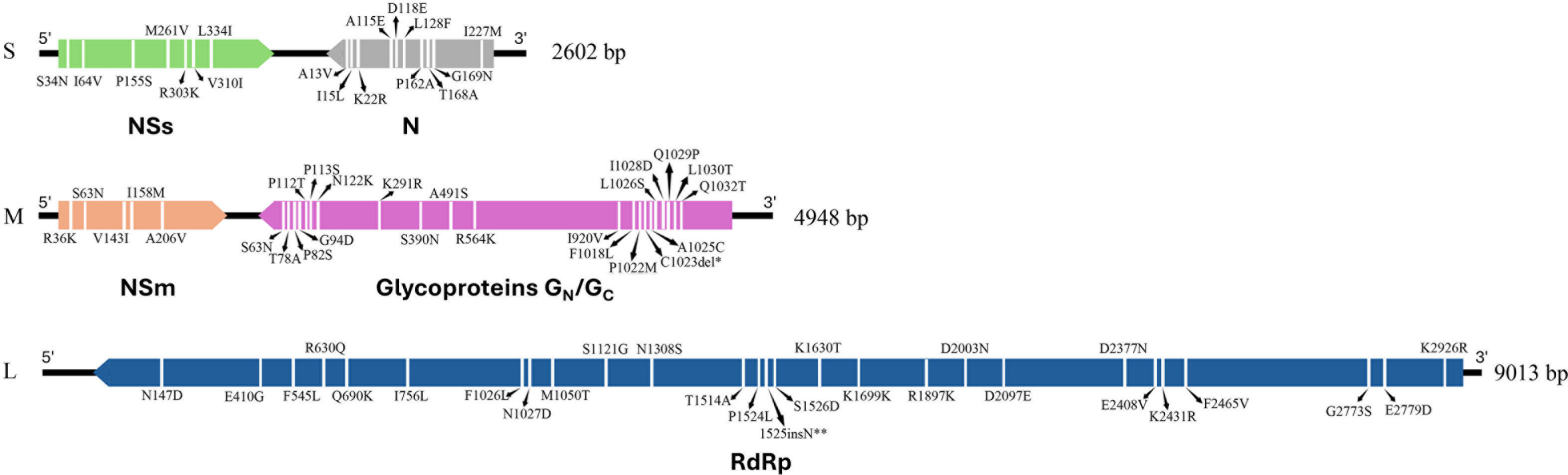
Amino acid mutations detected in the genome of the Alabama isolate SVNV17_Auburn_AL based on RNA-Seq data. Green indicates the nonstructural silencing suppressor protein (NSs), gray the nucleocapsid protein (N), orange the nonstructural movement protein (NSm), magenta the glycoproteins (G_N_ and G_C_), and blue the RNA-dependent RNA polymerase (RdRp). Asterisks (*) represent deletions; double asterisks (**) represent insertions. Tapered ends denote the direction of translation for each open reading frame (ORF).

The genome of the SVNV17_Auburn_AL comprises 16,563 bases (2,602 bp [S], 4,948 bp [M], and 9,013 bp [L]) with a GC content of 35% and an average depth of 1,669**×**. The leaders are 58, 57, and 185 bases, while the trailers are 70, 91, and 30 bases for the S, M, and L segments, respectively. The first six bases (AGAGCA) at the 5′ ends are identical across all three segments and are complementary to the 3′ ends, forming a panhandle similar to other orthotospoviruses (9). Genome comparison between the SVNV17_Auburn_AL isolate and the TN strain revealed 43, 97, and 138 SNPs/indels in the S, M, and L segments (Fig. 1). To determine the impact on the protein level, the ORFs’ sequence was translated with ExPASy (10) and aligned to the TN strain with Clustal Omega (11), identifying 17, 26, and 27 amino acid changes in the S, M, and L segment ORFs (3) (Fig. 1). Based on BLASTn (12), compared with the previous SVNV isolates from AL (AL-2 and AL-3) (S-MT669382.1, M-MT548010.1, and L-MT548011.1), our S, M, and L segments share 96%, 97%, and 98% nucleotide identity, indicating that this isolate is genetically distinct. Notably, our segments are more closely related to strains from Tennessee (NC_055178.1, 97.93%), Illinois (MT293139.1, 98.28%), and Iowa (MT536771.1, 98.78%), underscoring the genetic variability of SVNV across regions and highlighting the importance of continued genomic surveillance.

## Data Availability

The complete sequences of SVNV-AL in this paper are available at the NCBI under GenBank accession numbers: PV592793, PV592794, and PV592795. The SVNV-infected soybean RNA-Seq library sequence is under BioProject: PRJNA1246688, BioSample: SAMN47792314, and SRA: SRR32991661.

## AFTER EPUB

*[This article was published on 5 September 2025 with an error in the title. The title was corrected in the current version, posted on 15 September 2025.]*
